# Thrombotic Microangiopathy After Kidney Transplantation: Insights Into Genetic Etiology and Clinical Outcomes

**DOI:** 10.1016/j.ekir.2025.01.026

**Published:** 2025-01-30

**Authors:** Massini Merzkani, Nyein Chann Wai Lynn, Karen Flores, Gaurav Rajashekar, Kristin Progar, Rowena Delos Santos, John P. Atkinson, Daniel C. Brennan, Anuja Java

**Affiliations:** 1 Division of Nephrology, Department of Medicine, Washington University School of Medicine, St. Louis, Missouri, USA; 2 Division of Rheumatology, Department of Medicine, Washington University School of Medicine, St. Louis, Missouri, USA; 3 Division of Nephrology, The Johns Hopkins School of Medicine, Baltimore, Maryland, USA

**Keywords:** antibody-mediated rejection, calcineurin inhibitors, complement, genetics, kidney transplantation, thrombotic microangiopathy

## Abstract

**Introduction:**

Thrombotic microangiopathy (TMA), characterized by small-vessel thrombosis caused by endothelial injury, presents significant diagnostic and treatment challenges in kidney transplantation.

**Methods:**

To investigate the factors associated with posttransplant TMA, we conducted a retrospective study of 3535 kidney transplant recipients at our center from 2008 to 2023.

**Results:**

Sixty-eight patients were diagnosed with TMA, and 93% (63 of 68) underwent genetic testing. Patients were categorized into 3 groups based on the TMA etiology. Group 1 (*n* = 42, 62%) included patients with complement-mediated TMA associated with genetic or acquired complement abnormalities. These patients were younger and had a higher incidence of hypertension (HTN) or preeclampsia as the causes of end-stage kidney disease (ESKD). Notably, 33% of patients developed recurrent TMA, and approximately 78% of those with recurrent TMA lost their allografts. Group 2 (*n* = 14, 21%) had TMA associated with calcineurin inhibitors (CNIs) or ischemia-reperfusion injury (IRI), showing longer cold ischemia times (CITs) (19.9 ± 8.7 hours vs. 10.7 ± 8.9 hours; *P* = 0.0001) and a higher rate of delayed graft function (DGF) (43% vs. 13%, *P* = 0.0008) than the controls. Group 3 (*n* = 12, 17%) had TMA from diverse causes (infections or autoimmune disorders) and exhibited a poor response to anticomplement therapy. No genetic variants were identified in this group.

**Conclusion:**

Our findings underscore the need for comprehensive pre and posttransplantation genetic testing to predict and manage the risk of TMA and prevent graft loss. CNIs may exacerbate the risk of posttransplant TMA in the presence of other complement-activating factors. Our study also highlights the importance of personalized strategies and early interventions based on the functional assessment of variants of uncertain significance (VUS).

## Graphical abstract

**Figure:**
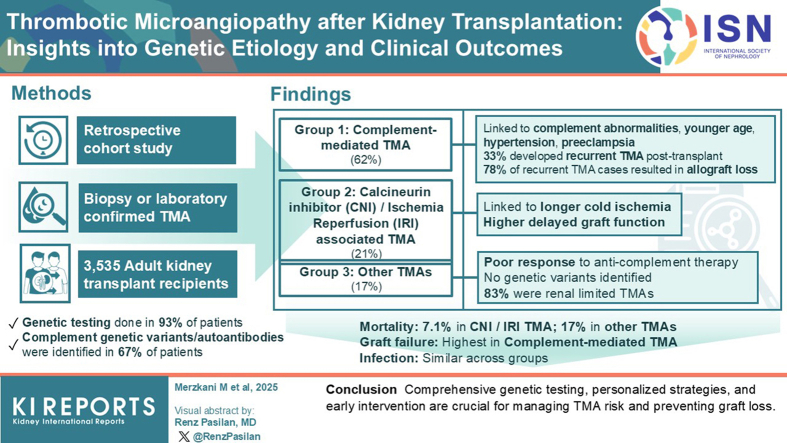

TMA is a complex clinicopathological entity characterized by microangiopathic hemolytic anemia, thrombocytopenia, and organ injury, which occurs because of endothelial damage and microthrombi formation in small vessels.^1^^,^^2^ Complement activation is a central mechanism underlying TMA, although its role varies in terms of onset and severity across different etiologies. TMA is classified as complement-mediated, if a genetic or acquired defect in a complement protein is identified as the driver of the disease. However, when TMA occurs in the context of other conditions such as infection, autoimmune disorders, or medications, further investigation should be undertaken to determine if the associated disease process is a trigger or driver of the disease because genetic abnormalities in the complement system may be identified in 10% to 60% of these patients.^3^^,^^4^ Efforts are ongoing to refine the TMA classification based on etiology and mechanism.^5^

In kidney transplant recipients, TMA affects up to 15% of the cases and poses diagnostic and therapeutic complexities because of diverse triggering factors.^6^^,^^7^ Posttransplant TMA can be recurrent (typically complement-mediated) or *de novo*,^8^^,^^9^ possibly triggered by medications, infections, rejection, and other factors. Distinguishing between complement-mediated TMA and TMA triggered by associated factors is critical for minimizing allograft damage, because the former requires targeted therapies and carries a higher risk of recurrence. A systematic approach is pivotal for accurate diagnosis and tailored treatment strategies.

At our center, we maintain a high index of suspicion for TMA in patients aged < 40 years with HTN-related ESKD, a history of preeclampsia or preexisting TMA, with the aim of conducting pretransplant testing for genetic and functional complement abnormalities in these individuals. This study evaluated over a decade of our center's experience with TMA in kidney transplant recipients, encompassing pretransplant evaluations, with > 90% having genetic testing, posttransplant diagnoses, and management strategies compared with those without posttransplantation TMA diagnoses.

## Methods

### Study Population

This retrospective cohort study examined adult kidney transplant recipients aged ≥ 18 years treated at Washington University School of Medicine (WUSM) in St. Louis, Missouri, USA, from January 2008 to February 2023. The study cohort included patients with either native kidney disease attributed to TMA or those diagnosed with “*de novo*” TMA posttransplantation. TMA diagnosis was confirmed by biopsy and/or a constellation of laboratory findings (hemoglobin < 10 g/dl, platelet count < 150,000/μl, serum lactate dehydrogenase > 1.5 times the upper limit of normal, undetectable haptoglobin, and acute kidney injury or DGF). All patients underwent ADAMTS13 activity and antiphospholipid antibody testing. Shiga toxin screening was conducted when clinically indicated to exclude typical hemolytic uremic syndrome (HUS). All allograft biopsies were systematically reviewed by a renal pathologist at the WUSM for 1 or more TMA-compatible histological features (mesangiolysis, fibrin thrombi in small arteries or glomerular capillaries, and arterial or arteriolar intimal edema/mucoid changes). The comparison cohort comprised of kidney transplant recipients from the same period at WUSM who did not develop TMA (also referred to as controls in this paper). This study was approved by the Institutional Review Board of the WUSM.

### Immunosuppression

Our standard induction regimen included thymoglobulin, which was used in 96% of the patients at doses ranging from 3 to 6 mg/kg depending on clinical requirements. In addition, all patients received intraoperative methylprednisolone (7 mg/kg, maximum 500 mg). Maintenance immunosuppressive therapy consisted of tacrolimus, prednisone, and either mycophenolate mofetil, or mycophenolate sodium. Patients experiencing gastrointestinal intolerance to mycophenolate transitioned to azathioprine. The tacrolimus target levels were aimed at 7 to 10 ng/ml during the initial month posttransplantation, with subsequent maintenance targeted at 4 to 7 ng/ml. The prednisone dose was tapered to 5 mg/d within the first month posttransplantation. Further details regarding each patient’s immunosuppressive regimen are presented in Supplementary Table S1.

### Data Collection

Data for TMA cases were collected through retrospective chart reviews from the WUSM electronic health records, the methodology and validity of which have been previously described.^10^^,^^11^ Data about the controls were obtained through retrospective reviews from the United Network of Organ Sharing database. Genetic analysis details were compiled for all patients who underwent testing conducted at WUSM's genomic and pathology services (gps.wustl.edu). We utilized a clinically validated next-generation sequencing assay for complement-mediated kidney diseases, employing the Agilent Clinical Research Exome capture reagent (Santa Clara, CA). This assay covers all exonic sequences of 15 genes (*ADAMTS13, C3, CD46, CFB, CFH, CFHR1, CFHR2, CFHR3, CFHR4, CFHR5, CFI, DGKE, THBD, MMACHC,* and *PLG*). Copy number variations in *CFHR3-CFHR1* were assessed using multiplex ligation-dependent probe amplification. Variants were reported by using the Human Genome Variation Society nomenclature and classified according to joint guidelines from the American College of Medical Genetics and the Association for Molecular Pathology, categorizing variants as pathogenic, likely pathogenic, VUS, likely benign, or benign.^12^

### Outcomes Measured

The outcomes assessed included the following: incidence of recurrent TMA (based on laboratory parameters or biopsy confirmation, when feasible), time to TMA onset posttransplantation, type of TMA presentation (systemic vs. renal-limited), frequency of infections such as cytomegalovirus (CMV), BK virus, urinary tract infections, other infections requiring hospitalization, occurrence of acute rejection episodes, death-censored graft failure, and death with functional allografts. Death-censored graft failure was defined as return to chronic dialysis or receiving another kidney transplant, whereas death with a functional allograft was defined as mortality before graft failure.

### Statistical Analysis

Data were reported as mean (± SD) or median (interquartile range) based on normality of distribution, whereas categorical data were presented as percentages. Continuous clinical characteristics were compared using *t* tests, and chi-square tests were used for categorical variables. Data were censored at the patients’ last follow-up or in April 2023. All statistical analyses were 2-sided, with significance set at *P* ≤ 0.05. The incidences of death with functional allografts and death-censored graft failure were modeled using univariate and multivariate Cox regression analyses. The risk of recurrence was assessed by using univariate and multivariate logistic regression analyses. Statistical analysis was performed using JMP PRO v.15 software (www.jmp.com).

## Results

### Patient Demographics

We identified 68 patients with TMA at WUSM between January 2008 and February 2023 (Table 1). Only 2 patients were identified between 2008 and 2012 and the remaining were identified between 2012 and 2023. The mean follow-up duration was 3.6 ± 2.8 years. The recipients had a mean age of 43.8 ± 15.3 years, with 50% being male, 68% White, and 28% Black. The majority (82%) underwent deceased donor kidney transplantation, and 19% had a previous kidney transplantation. The average CIT for the cohort was 12.3 ± 9.6 hours. Genetic variants in or acquired autoantibodies to complement proteins were identified in 67% of the patients (based on testing conducted in 63 of 68 patients).

**Table 1:** Baseline characteristics of thrombotic microangiopathy cohort (*n* = 68)

Variable	Mean ± SD or *n* (%)
Recipient characteristics	
Age (yr)	43.8 ± 15.3
Sex	
• Males	34 (50%)
• Females	34 (50%)
Race	
• White, Non-Hispanic	46 (67.6%)
• Black, Non-Hispanic	19 (27.9%)
• Asian, Non-Hispanic	2 (2.9%)
• Hispanic/Latino	1 (1.5%)
BMI (kg/m^2^)	28.0 ± 7.0
Previous kidney transplant	13 (19.1%)
Pretransplant dialysis	61 (89.7%)
Preformed DSA	14 (20.6%)
CMV seropositive (IgG+)	36 (52.9%)
Type of donor	
• Deceased donor	56 (82.4%)
• Living related	3 (4.4%)
• Living unrelated	9 (13.2%)
Genetic variant/autoantibody identified	42 (66.7%; 42/63)
Donor characteristics	
Age (yr)	36.1 ± 15.9
Sex (males)	45 (66.2%)
Race	
• White, Non-Hispanic	57 (83.9%)
• Black, Non-Hispanic	8 (11.9%)
• Asian, Non-Hispanic	1 (1.5%)
• Hispanic/Latino	2 (3%)
BMI (kg/m^2^)	28 ± 7.7
Transplant characteristics	
Cold ischemia time (h)	12.3 ± 9.6
Kidney donor profile index (KDPI; %)	32.6 ± 26.7
Delayed graft function	18 (27.3%)
HLA mismatch	4.0 ± 1.4
Induction immunosuppression	
• Thymoglobulin	65 (95.6%)
• Basiliximab	2 (2.9%)
• Campath	1 (1.5%)

BMI, body mass index; CMV, cytomegalovirus; DSA, donor-specific antibody; HLA, human leukocyte antigen.

The comparison or control cohort comprised 3467 kidney transplant recipients between 2008 and 2023 who did not develop TMA in the allograft (Table 2). These recipients had a mean age of 53.2 ± 14.3 years, with 61% being male and 68% White. Among them, 74% received a deceased donor transplant, and 11% had a previous kidney transplant. The average CIT was 10.7 ± 8.9 hours.

**Table 2:** Baseline characteristics of the control cohort (*n* = 3467)

Variable	Mean ± SD or n (%)
Recipient Characteristics	
Age (yr)	53.2 ± 14.3
Sex	
• Males	2109 (60.8%)
• Females	1358 (39.2%)
Race	
• White, Non-Hispanic	2366 (68.2%)
• Black, Non-Hispanic	930 (26.8%)
• Others	171 (5%)
BMI (kg/m^2^)	28.9 ± 5.8
Previous kidney transplant	393 (11.3%)
Pretransplant dialysis	2509 (72.4%)
CMV seropositive (IgG+)	2083 (60.1%)
Type of donor	
• Deceased donor	2553 (73.6%)
• Living related	441 (12.7%)
• Living unrelated	473 (13.6%)
Donor characteristics	
Age (yr)	39.1 ± 15.2
Sex (Males)	1890 (54.5%)
Race	
• White, Non-Hispanic	2866 (82.7%)
• Black, Non-Hispanic	445 (12.8%)
• Others	156 (4.5%)
BMI (kg/m^2^)	31.4 ± 14.1
Transplant characteristics	
Cold ischemia time (h)	10.7 ± 8.9
KDPI (%)	39.3 ± 24.9
Delayed graft function	445 (12.8%)
HLA mismatch	3.8 ± 1.6

BMI, body mass index; CMV, cytomegalovirus; HLA, human leukocyte antigen; KDPI, Kidney Donor Profile Index.

### Genetic Results and Interpretation

Genetic testing was performed in 63 of the 68 patients (93%) in the cohort (Supplementary Table S1). Among these, 13 patients (21%) were found to carry known pathogenic or likely pathogenic mutations, 2 patients had benign or likely benign variants, and VUS was initially identified in 17 patients (27%). Subsequent functional studies conducted by our laboratory and others demonstrated that 12 of these VUS (∼70.5%) were deleterious.^13^, ^14^, ^15^, ^16^, ^17^, ^18^, ^19^, ^20^, ^21^, ^22^, ^23^ In addition, 10 patients tested positive for factor H autoantibody, with 7 associated with a homozygous *CFHR3-1* deletion. Genetic variants were not detected in 21 patients. Among the remaining 5 patients who did not undergo genetic testing, 2 lacked clear reasons, whereas a genetic etiology was not considered for the other 3 who promptly responded to the cessation of CNI therapy.

Based on our findings, the patients were classified into 3 groups (Table 3). Group 1 comprised individuals with identified genetic variants or acquired autoantibodies in the complement genes, diagnosed with complement-mediated TMA either before or after transplantation; 42 of the 68 patients belonged to this group. Twelve patients had a biopsy-proven diagnosis of TMA, whereas the remaining patients were diagnosed based on laboratory criteria. Group 2 consisted of 14 patients who developed TMA associated with CNI usage that likely exacerbated IRI. Group 3 included 12 patients with TMA associated with other causes such as viral infections or autoimmune diseases. Two patients in group 2 and 6 patients in group 3 had a biopsy-proven diagnosis of TMA, whereas the remaining patients were diagnosed based on laboratory features.

**Table 3:** Comparison of the baseline characteristics of the 3 TMA groups with controls

Demographics	Controls *n* = 3467	Group 1 (complement-mediated TMA) *n* = 42	*P*-value (group 1 vs. controls)	Group 2 (CNI/IRI-associated TMA) *n* = 14	*P*-value (group 2 vs. controls)	Group 3 (other TMA) *n* = 12	*P*-value (group 3 vs. controls)
Recipient characteristics							
Age (yr)	53.2 ± 14.3	38.1 ± 11.4	< 0.0001	55.1 ± 15.2	0.61	51.1 ± 18.3	0.6
Males, *n* (%)	2109 (60.8%)	23 (54.8%)	0.42	7 (50%)	0.41	4 (33.3%)	0.05
Race, *n* (%)							
White Non-Hispanic	2366 (68.2%)	29 (69.0%)	0.99	8 (57.2%)	0.7	9 (75%)	0.7
Black Non-Hispanic	930 (26.8%)	11 (26.2%)		5 (35.7%)		3 (25%)	
Others (Asian/Hispanic)	171 (5 %)	2 (4.8%)		1 (7.1%)		0 (0%)	
BMI (Kg/m^2^)	28.9 ± 5.8	29.3 ± 7.5	0.6	27.5 ± 5.8	0.35	28.8 ± 6.5	0.98
Previous kidney transplant n (%)	393 (11.3%)	12 (28.6%)	0.0005	1 (7.1%)	0.62	0 (0%)	0.21
Pretransplant dialysis, *n* (%)	2509 (72.4%)	37 (88.1)	0.02	12 (85.7%)	0.26	11 (91.7%)	0.13
Type of donor, *n* (%)							
• Deceased donor	2553 (73.6%)	30 (71.4%)	0.75	14 (100%)	0.02	12 (100%)	0.03
• Living related	441 (12.7%)	3 (7.1%)	0.28	0 (0%)	0.15	0 (0%)	0.19
• Living unrelated	473 (13.6%)	9 (21.4%)		0 (0%)		0 (0%)	
Tacrolimus trough (ng/ml; mean ± SD)	NA	7.5 ± 2.8	NA	6.5 ± 5.7	NA	9.0 ± 3.7	NA
Donor characteristics							
Age (yr)	39.1±15.2	33.2±14.3	0.01	39.3±18.3	0.97	42.8±17.1	0.39
Males, *n* (%)	1890 (54.5%)	28 (66.7%)	0.11	10 (71.5%)	0.2	7 (58.3%)	0.8
Race							
• White, Non-Hispanic	2866 (82.7%)	35 (83.3%)	0.98	12 (85.7%)	0.71	10 (83.3%)	0.75
• Black, Non-Hispanic	445 (12.8%)	5 (11.9%)		2 (14.3%)		1 (8.3%)	
• Others	156 (4.5%)	2 (4.8%)		0 (0%)		1 (8.3%)	
BMI (kg/m^2^)	31.4 ± 14.1	28.7±7.3	0.23	28±8.7	0.37	26.5±6.4	0.23
Transplant characteristics							
Cold ischemia time (hr)	10.7 ± 8.9	10.7 ± 9.3	0.96	19.9 ± 8.7	0.0001	9.2 ± 6.8	0.56
KDPI (%)	39.3 ± 24.9	22.8 ± 21.4	0.0003	46.3 ± 29.8	0.29	41.4 ± 26.8	0.76
DGF, *n* (%)	445 (12.8%)	8 (19%)	0.3	6 (42.9%)	0.0008	4 (33.3%)	0.03
HLA mismatch	3.8 ± 1.6	3.9 ±1.4	0.54	4.0 ± 1.5	0.49	4.3 ±1.6	0.3

BMI, body mass index; CNI, calcineurin inhibitor; DGF, delayed graft function; HLA, human leukocyte antigen; IRI, ischemia-reperfusion injury; KDPI, Kidney Donor Profile Index; TMA, thrombotic microangiopathy.

#### Group 1 (Complement-Mediated TMA; 42 Patients)

Patients in the complement-mediated TMA group were significantly younger than those in the comparison or control cohort (38.1 ± 11.4 years vs. 53.2 ± 14.3 years, *P* < 0.0001) (Table 3). Similarly, recipients in this group were younger than both those in group 2 (38.1 ± 11.4 years vs. 55.1 ± 15.2 years, *P* < 0.0001) and those in group 3 (38.1 ± 11.4 years vs. 51.1 ± 18.3 years, *P* = 0.004) (Tables 3 and 4). A higher proportion of patients in this group had a history of previous kidney transplant (28.6% vs. 11.3%, *P* = 0.0005) and received kidneys with a lower Kidney Donor Profile Index (22.8 ± 21.4 vs. 39.3 ± 24.9, *P* = 0.0003) than the controls (Table 3). The mean tacrolimus level at the time of TMA presentation was 7.5 ± 2.8 ng/ml. The median time to TMA development after transplantation was 7 days. Approximately 43% (18 of 42) of the patients presented with renal-limited TMA, defined as TMA identified solely on kidney biopsy without hematological manifestations.

**Table 4:** Comparison of the baseline characteristics of the 3 TMA groups

Demographics	Group 1 vs. Group 2	Group 1 vs. Group 3	Group 2 vs. Group 3
	*P*-value	*P*-value	*P*-value
Age (yr)	< 0.0001	0.004	0.54
Males	0.75	0.19	0.39
BMI (Kg/m^2^)	0.37	0.83	0.56
Race	0.71	0.73	0.49
Previous kidney transplant (%)	0.1	0.03	0.34
Pretransplant dialysis (%)	0.81	0.73	0.63
Type of donor			
Deceased	0.02	0.03	NA
Living (related vs unrelated)	0.3	0.34	NA
TMA presentation			
Systemic vs. renal limited	0.01	0.01	0.0001
Mean tacrolimus trough (ng/ml)	0.25	0.12	0.04
Cold ischemia time (h)	0.002	0.58	0.002
KDPI (%)	0.005	0.02	0.67
Delayed graft function (%)	0.07	0.29	0.62

BMI, body mass index; KPDI, kidney donor profile index; TMA, thrombotic microangiopathy.

Among the 42 patients in this group, 8 (19%) had a preexisting diagnosis of atypical HUS (aHUS) before transplantation (Supplementary Table S1). Seven patients (∼17%) had “HUS,” or “biopsy-proven TMA” reported as the cause of ESKD. Twenty-one patients (50%) had hypertensive nephrosclerosis or preeclampsia as the cause of ESKD. Other etiologies of ESKD included lupus or IgA nephropathy in 4 patients, and membranous nephropathy and autosomal dominant polycystic kidney disease, each identified in 1 patient. Notably, several variants in group 1 (#9, #13, #15, #17, #18, #24, #33, #35, #37, #38, #41, and #42) were reported as VUS at the time of testing; however, subsequent functional studies by us and others characterized these variants as deleterious. The references are cited next to the variants in Supplementary Table S1. Two patients in group 1 carried a VUS that was not functionally characterized (#25 and #39) because of the challenges of recombinantly making these variants; 2 patients did not undergo genetic testing for unclear reasons (#23 and #36). Because of their clinical course in the native disease, recurrence after kidney transplant, and loss of allografts to thrombosis and TMA, these cases were highly concerning for a complement-mediated process. Therefore, these patients were included in group 1.

Detailed information on the identified genetic variants, their interpretations, current immunosuppressive regimens, outcomes of previous allografts (for those with multiple transplants), and latest creatinine and proteinuria levels as of April 2023 are provided in Supplementary Table S1.

Thirty-two of the 42 patients (76%) were treated with a complement inhibitor (eculizumab or switched to ravulizumab based on patient preference). The remaining 10 patients either returned to dialysis or died. Twenty-three of the 32 patients were treated with eculizumab at the time of transplantation because of a known history of complement-mediated TMA (either based on native kidney disease or identified by genetic testing during the pretransplant evaluation). Nine patients began treatment after TMA was diagnosed in the allografts. The mean duration of anti-C5 treatment was 4.2 ± 2.9 years. Anticomplement therapy was discontinued in 5 patients after varying periods (3 months–2 years) posttransplantation. Among these patients, 4 had an undetectable factor H autoantibody at the time of drug cessation and 1 had a VUS in the membrane cofactor protein. All individuals receiving complement inhibitors were vaccinated and maintained on prophylactic antibiotics to prevent infection by encapsulated organisms.

Fourteen of the 42 patients (33.3%) experienced recurrent TMA (Table 5 and Supplementary Table S1), with a median time to recurrence of 7 days. Two of the 14 patients (patients #11 and #27) were diagnosed with TMA in the native kidney. However, the other 12 patients (#15, #17, #19, #21–#23, #35–#38, #41, and #42) were initially considered *de novo* (because the native disease included HTN, preeclampsia, and lupus), and several of these patients were switched from tacrolimus to belatacept-based immunosuppression, but cessation of CNI did not resolve the TMA. Subsequent genetic testing revealed complement mutations. Therefore, these cases were likely recurrent and undiagnosed in the native kidneys. After these patients were identified and treated with anticomplement therapy, they responded with no further allograft recurrence. Alarmingly, 11 of the 14 patients (78.5%) lost an allograft because of TMA. These patients were not on anticomplement therapy at the time because the diagnosis of complement-mediated TMA was established after recurrence in the allograft. Five patients (12%) in group 1 experienced T-cell–mediated rejection or antibody-mediated rejection (AMR). No cases of meningococcal or pneumococcal infections were reported (Table 5). The incidences of other infections (CMV, BK viremia, and urinary tract infections) in this group are shown in Table 5. Death-censored graft failure occurred in 26% of patients, and death with a functioning graft occurred in 9.5% of patients. Multivariate analysis for death-censored graft failure indicated a higher risk associated with younger age (adjusted hazard ratio = 0.88 [0.79–0.96]; *P* = 0.01) and human leukocyte antigen A mismatch (adjusted hazard ratio= 4.42 [1.4–18.0]; *P* = 0.02) (Supplementary Table S2). However, the characteristics associated with a higher risk of death with a functioning graft or recurrent TMA were not identified in the multivariate analyses (Supplementary Tables S3 and S4).

**Table 5:** Comparison of measured outcomes for the 3 TMA groups

Measured outcomes	Group 1 (*n* = 42)	Group 2 (*n* = 14)	Group 3 (*n* = 12)	
	*n* (%)	*n* (%)	*n* (%)	*p*-value
Recurrent TMA	14 (33.3%)	None	None	
Median time to TMA development/recurrence after transplant (days)	7	5	455	0.002
TMA presentation				
• Systemic	24 (57.1%)	13 (92.8%)	2 (16.7%)	0.0005
• Renal limited	18 (42.8%)	1 (7.2%)	10 (83.3%)	
Switched to belatacept	5 (11.9%)	12 (85.7%)	2 (16.7%)	<0.0001
Eculizumab	32 (76.2%)	2 (14.3%)	2 (16.7 %)	0.001
CMV infection	2 (4.8%)	2 (14.5%)	5 (41.7%)	0.004
BK infection	9 (21.4%)	2 (14.3%)	2 (16.7%)	0.82
Urinary tract infection	6 (14.3%)	4 (28.6%)	4 (33.3%)	0.25
Meningococcal infection	0 (0%)	0 (0%)	0 (0%)	
Pneumococcal infection	0 (0%)	0 (0%)	1 (8.3%)	0.09
Other infections requiring hospitalization	5 (11.9%)	6 (42.8%)	4 (33.3%)	0.03
Death-censored graft failure	11 (26.2%)	1 (7.1%)	2 (16.7%)	0.32
Death with functioning graft	4 (9.5%)	1 (7.1%)	2 (16.7%)	0.49

CMV, cytomegalovirus; TMA, thrombotic microangiopathy.

#### Group 2 (IRI/CNI-Associated TMA; 14 Patients)

All the patients in this group developed TMA after kidney transplantation. None of these patients had a history of TMA or HUS as the cause of native kidney disease and therefore developed *“de novo”* TMA with a median time to TMA development after transplant of 5 days. In this group, patients experienced TMA, which were likely caused by IRI associated with CNI use. They exhibited significantly longer CIT than the controls (19.9 ± 8.7 vs. 10.7 ± 8.9, *P* = 0.0001) (Table 3) and a markedly higher incidence of DGF than the controls (43% vs. 13%, *P* = 0.0008) (Table 3). Furthermore, patients in this group had longer CIT than group 1 (19.9 ± 8.7 vs. 10.7 ± 9.3, *P* = 0.002) and group 3 (19.9 ± 8.7 vs. 9.21 ± 6.8, *P* = 0.002) (Table 4). The mean tacrolimus level at the time of TMA diagnosis was 6.5 ± 5.7 ng/ml for this cohort. One patient presented solely with renal-limited TMA, whereas the remaining patients (13/14; 93%) had systemic hematological manifestations.

Genetic testing was conducted in 11 of the 14 patients (#43–#56; Supplementary Table S1). Nine patients carried no genetic variants or factor H autoantibodies. In 12 patients, tacrolimus was switched to belatacept, whereas 1 patient was switched to everolimus (because Epstein-Barr virus negativity precluded belatacept use). Tacrolimus was continued in 1 patient along with eculizumab because of a family history of thrombocytopenia that raised concerns about a genetic etiology, prompting eculizumab initiation instead of a belatacept switch. Subsequent genetic testing revealed a heterozygous VUS in *DGKE*; however, given that *DGKE* mutations linked to aHUS typically manifest at an early age, are homozygous, and do not respond to anticomplement therapy, we speculated that a heterozygous variant in the 59-year-old was not the underlying cause of TMA and eculizumab was discontinued without any recurrence.

Three patients resumed tacrolimus later in their transplantation journey for various reasons after TMA resolution (Supplementary Table S1). Importantly, none of these patients experienced TMA after CNIs were reintroduced, supporting the concept that CNIs likely exacerbate the risk of IRI and TMA in the early postoperative period. Among the 14 patients, 2 developed acute cellular rejection and/or AMR. No cases of meningococcal or pneumococcal infections were reported (Table 5). The incidences of CMV, BK viremia, and urinary tract infections did not significantly differ between the groups (Table 5). One patient returned to dialysis and another died, resulting in calculated incidence of 7.1% each for death-censored graft failure and death with a functioning graft. Owing to the small sample size of this group, neither univariate nor multivariate analyses were conducted.

#### Group 3 (TMA-Associated With Other Etiologies; 12 Patients)

Patients in group 3 developed TMA associated with various etiologies (#57–#68; Supplementary Table S1). Their baseline characteristics and CITs were similar to those of the controls; however, the rate of DGF was slightly higher than the controls (*P* = 0.03) (Table 3). The mean tacrolimus level was 9 ± 3.7 ng/ml. Most patients (83%) presented with renal-limited TMA. No genetic variants were identified in any of the patients. Six recipients had a history of TMA or HUS as the cause of native kidney disease but did not experience recurrence. Another 6 patients developed “*de novo*” TMA posttransplantation, with a median time to TMA development of 455 days. In 3 patients, TMA did not respond to tacrolimus cessation or anticomplement therapy (despite total hemolytic complement activity [CH50] < 10 units/ml). The incidence of CMV viremia was higher in this group than in the other 2 groups (*P* = 0.004) (Table 5). Four of 12 patients developed acute cellular rejection and/or AMR. One patient had a pneumococcal infection, but did not receive anticomplement therapy. Two patients returned to dialysis and 2 died, resulting in a 16.7% incidence of death-censored graft failure and death with a functioning graft. Owing to the small cohort size, neither univariate nor multivariate analyses were conducted for this group.

## Discussion

In this study, we conducted a retrospective review of 68 kidney transplant recipients from 2008 to 2023 diagnosed with TMA, 93% of whom had genetic testing, either pre- or posttransplant, to comprehensively assess etiology and outcomes. We compared these cases with 3467 transplant recipients during the same period (50:1 ratio). Our study stratified the patients into 3 groups: complement-mediated TMA (group 1, 42 patients), IRI/CNI-associated TMA (group 2, 14 patients), and TMA associated with other etiologies (group 3, 12 patients), yielding several key findings.

First, half of the patients with complement-mediated TMA (21 of 42) had a history of HTN or preeclampsia as a cause of ESKD. Among these, 14 of 42 (33%) developed recurrent TMA (12 of the 14 were initially thought to be “*de novo*” TMA but identification of complement mutations established that these were recurrent and not *de novo*). Eleven of these 14 patients (78.5%) with recurrent TMA experienced graft loss shortly after kidney transplantation. These results underscore the importance of comprehensive pretransplant evaluation for complement abnormalities in patients with known aHUS or biopsy-proven TMA. In addition, young patients (mean age 38.1 ± 11.4 years based on our data) progressing to ESKD caused by hypertensive nephrosclerosis, preeclampsia, or another aggressive form of “secondary TMA” should undergo genetic testing as part of their pretransplant assessment.

Second, genetic testing was performed in approximately 93% of the patients (63 of 68), which we believe represents one of the largest kidney transplant cohorts with comprehensive complement genetic testing to-date. Notably, 17 of 63 patients were initially reported to have a VUS at the time of testing; however, 12 of the 17 VUS (70.5%) have since been characterized by us and others to be deleterious based on antigenic or structure-function analyses, providing critical insights into disease etiology.^13^, ^14^, ^15^, ^16^, ^17^, ^18^, ^19^, ^20^, ^21^, ^22^, ^23^ This process helped guide decisions regarding the need for prophylactic eculizumab or lifelong treatment, facilitating personalized therapeutic approaches based on individual risk assessments. In addition, our findings indicate that in 4 of 10 patients (40%), factor H autoantibodies decreased over time after immunosuppression, allowing for the discontinuation of anticomplement therapy with vigilant monitoring for recurrence. Furthermore, patients (group 3) who were diagnosed with TMA in the native kidney but did not harbor genetic mutations, experienced no recurrence after transplantation.

Third, a small subset of patients (4 of 42) in the complement-mediated TMA group presented with AMR on allograft biopsy, which is a notable finding given that AMR is considered a potential confounder for TMA. This association has been previously documented by other researchers.^24^^,^^25^ Recently, a study by Dessaix *et al.* identified AMR and CNI as the 2 primary causes of *de novo* TMA posttransplantation.^26^ This issue has sparked debate within the TMA Banff working group, highlighting the challenge of distinguishing between AMR and TMA, and whether AMR can potentially induce TMA.^27^ Based on these observations, we hypothesize that in certain patients, AMR might resemble TMA more closely than currently appreciated, necessitating further systematic investigation in future studies.

Fourth, patients with IRI/CNI-associated TMA were older (mean age 55.1 ± 15.2 years) and experienced higher rates of DGF and longer CIT than the controls and other 2 groups. Interestingly, the mean tacrolimus trough levels were comparable across all groups, suggesting that CNIs may exacerbate the risk of IRI in the early posttransplantation period, irrespective of the dosage or trough levels. Notably, 3 of the 14 patients were subsequently reintroduced to CNI therapy without experiencing recurrent TMA. Therefore, at this time, the role of CNI alone in causing TMA cannot be fully elucidated. A limitation of this analysis was the small sample size in the CNI-associated TMA group, necessitating ongoing vigilant monitoring, particularly as emerging evidence implicates complement dysregulation in CNI-related endothelial injury.^28^^,^^29^

Fifth, group 3 in our study represents an intriguing yet challenging cohort. No complement genetic variants were detected. The time to TMA development after transplantation varied widely, with some patients presenting several months to years later (median time to TMA development was 455 days), in contrast to groups 1 and 2, which manifested early posttransplantation (within 5–7 days). The wide difference in the time to TMA development is likely reflective of the underlying etiology. Patients in group 1 had underlying complement dysregulation caused by genetic mutations, whereas patients in group 2 likely had endothelial injury caused by IRI and/or CNI, leading to an early presentation after a complement-amplifying event of transplant surgery. These patients responded to anti-complement therapy or holding the CNI is the immediate post-transplant period respectively. However, group 3 was a heterogeneous group that developed TMA caused by factors that were not entirely because of complement activation. Anticomplement therapy and cessation of CNIs showed limited efficacy in this group. The number of patients in group 3 presenting with acute or chronic features on biopsy (such as thrombi, mesangiolysis, interstitial fibrosis, and tubular atrophy) was comparable to that in the other groups. Although only a subset of patients underwent kidney biopsy, and larger numbers are needed to validate these findings, they emphasize the multifaceted nature of the etiology of endothelial injury and the unresolved role of complement activation in this group.

Sixth, a subset of patients in each group presented with renal-limited TMA (18 of 42 [43%] in group 1, 14 in group 2, and 10 of 12 [83%] in group 3). However, the true incidence of renal-limited TMA may have been underestimated because of the varying rates of renal biopsy. Previous studies have reported renal-limited TMA in both native and posttransplant kidneys, with reported incidences ranging from 38% to 45%, leading to diverse outcomes.^7^^,^^30^, ^31^, ^32^ The underlying pathophysiological mechanism explaining why some patients exhibit classic systemic findings whereas others present with renal-limited TMA remains unknown. Proposed mechanisms beyond complement activation exist but lack definitive evidence.^33^

Our study has several limitations. First, our comparison or control cohort, sourced from the United Network of Organ Sharing database, lacked detailed information on the immunosuppression regimens used. Nonetheless, based on our center’s protocol, it is anticipated that a majority of patients received thymoglobulin and steroids for induction, alongside our standard maintenance regimen comprising tacrolimus, mycophenolate, and prednisone. Second, in group 1, there were 2 patients in whom the significance of a VUS could not be fully elucidated and 2 who did not undergo genetic testing; however, based on the clinical course of disease in the native kidney, recurrences after kidney transplant and loss of allografts to thrombosis and TMA, these cases were highly concerning for a complement-mediated process. Third, there were 2 patients in group 1 who carried *THBD* variants. *THBD* was first reported in relation to aHUS in 2009;^34^ however, *THBD* mutations have recently been controversial because published variants in *THBD* have a high minor allele frequency for rare diseases such as aHUS, and enrichment analysis has not validated the association of *THBD* with aHUS. Therefore, our working group at ClinGen recently refuted the association between *THBD* and aHUS (https://clinicalgenome.org/affiliation/40069/). However, other group curations, such as those by Genomenon (https://mastermind.genomenon.com/gene?gene=thbd&gene_op=and&utm_source=dan&utm_medium=email&utm_campaign=1009_thbd_basic) and Invitae (https://www.invitae.com/us/providers/test-catalog/gene-200989), have shown a strong association between *THBD* and aHUS.

Therefore, this remains an area that needs to be clarified further. Of the 2 patients in group 1 who carried *THBD* variants, one had a native diagnosis of HUS and developed recurrent TMA in the allograft, and the other patient carried a variant of *THBD* (C224G), which is novel and rare, with a very low allele frequency of 6.62 × 10^−7^ (unlike other published *THBD* variants). Therefore, at this time, we are treating these patients with anti-C5 to prevent TMA in the allograft and have left them in group 1. As more data on *THBD* variants become available and the controversy is settled, we will consider discontinuing this patient from anti-C5 therapy and monitoring. Fourth, because of the small number of patients in groups 2 and 3, we were unable to perform multivariate analyses for these cohorts. Notably, our multivariate analysis of group 1 revealed a heightened risk of death-censored graft failure associated with human leukocyte antigen A mismatch. Although this finding is novel, its exact significance remains to be fully understood. Fifth, this was a retrospective chart review of patients between 2008 and 2023; however, we identified only 2 patients diagnosed with TMA between 2008 and 2012. Because this was the time period before the approval of anticomplement therapy and the understanding of complement and TMAs was evolving, it is possible that the patients were underdiagnosed. The use of specific pathologies and laboratory findings in the inclusion criteria may have also led to missing cases.

Two recent informative studies are relevant to our work. In a study by Tokarski *et al.*, TMA presentations were classified as early (linked with anti–human leukocyte antigen class II antibodies, preformed donor-specific antibody, and acute AMR with a favorable prognosis), unexpected late (associated with viral infections, chronic AMR, and CNI use), or resulting in graft failure (associated with malignant HTN, AMR, and infections).^24^ Our findings expand and clarify the etiology of these presentations. In our study, most patients who presented early or experienced graft loss caused by TMA had complement-mediated TMA, which is often undiagnosed before transplantation. Unlike the Tokarski study, patients with CNI-associated TMA in our cohort presented early and responded well to drug cessation, with no recurrence upon reintroduction, highlighting the impact of multiple triggers in the early posttransplant phase like ischemia-reperfusion. Patients with unexpected late TMA in the Tokarski study resembled those in group 3, in which TMA was linked to AMR and CMV viremia. In another study by Maritati *et al.*, early use of eculizumab correlated with improved graft outcomes.^35^ Their study, involving a significant proportion of extended-criteria donors and limited genetic findings, opted to continue CNIs alongside anticomplement therapy because of the absence of belatacept. Posttransplant TMA in this cohort likely stemmed from CNI use and IRI. Their approach to early short-term eculizumab use along with ongoing CNIs appears appropriate within their context.

Overall, our study provided a comprehensive assessment of the etiology and outcomes of TMA after kidney transplantation at our center. This underscores the critical role of complement genetic testing in young patients during pretransplant evaluation and how overlooking complement dysregulation can precipitate early posttransplant recurrence. Our work emphasizes that a systematic approach is crucial for an accurate diagnosis and enables personalized treatment decisions for patients. It also highlights that TMA after kidney transplantation can occur because of etiologies other than complement dysregulation. These other forms of TMA are often renal-limited and respond poorly to complement inhibition, likely because of noncomplement causes of endothelial injury. The exact management of these patients remains unclear, underscoring the research gaps that warrant further investigation.

Until such research clarifies unanswered questions, one strategy that we currently use in our practice to better evaluate and treat posttransplant TMA patients, involves identifying “high risk” patients during preevaluation. These patients include those with a personal or family history of TMA and young patients who have developed ESKD caused by HTN, preeclampsia, or unknown causes. These patients undergo genetic testing to evaluate complement defects. If a pathogenic mutation is identified in a complement gene, which is clear in its ability to cause dysregulation of complement activation, prophylactic anticomplement therapy is considered at the time of transplantation and continued thereafter. If a VUS is identified, further work-up (including antigenic and/or functional testing) is conducted to determine the significance of the variant and to decide the prophylaxis and duration of treatment. Given that this workup is conducted during preevaluation, there is sufficient time to make an informed decision about the risk of developing posttransplant TMA and to plan posttransplant management.

If workup for a “high-risk” patient is missed during pretransplant evaluation and the patient develops TMA early posttransplant, our suspicion for a complement-mediated process is high and we start anticomplement therapy as soon as possible (without necessarily stopping the CNI). Genetic and autoantibody testing for complement is requested, and once the results are available (approximately 4–6 weeks later), a decision regarding continuing or discontinuing anti-C5 treatment is made. If a pathogenic mutation is identified, treatment is continued indefinitely. VUSs are further investigated to determine their significance. If genetic testing is negative and kidney function has recovered, the anti-C5 antibody is discontinued and the patient is monitored closely. If genetic testing is negative and kidney function has not recovered, we may consider continuing anti-C5 therapy longer (maybe 6–12 months) but would eventually consider stopping based on a negative genetic etiology. If TMA recurs, anti-C5 therapy is restarted, and other etiologies (such as CNI, other medications, viral infections, AMR etc.) are investigated and treated. In the absence of an identified etiology, such patients may need to stay on therapy indefinitely (and likely belong to ∼30% of patients with complement-mediated TMA without an identified genetic etiology). Patients who lose an allograft in the posttransplant setting to TMA are administered prophylactic anticomplement therapy at the time of the next transplant (even in the absence of a genetic mutation) and continued on it indefinitely.

For older patients (aged > 50 years) who develop posttransplant TMA and where the native disease is not related to a complement-mediated process, we hold the CNI (and replace it with belatacept), request for genetic testing, test for other etiologies (viral infections, metabolic etiologies, monoclonal gammopathy, etc.), and continue monitoring. If there is no response to holding the CNI and no other etiology is identified, anti-C5 is initiated. If genetic testing returned negative results and kidney function has recovered, the anti-C5 is discontinued and monitored. Often, these cases are because of long CIT and IRI that lead to endothelial damage in the presence of CNI. Reviewing the progress of the mate kidney in such cases may be helpful, if available. If these patients develop recurrent TMA, additional etiologies are investigated, and anti-C5 therapy is restarted and continued indefinitely.

## Disclosure

AJ serves on scientific advisory boards of Alexion, AstraZeneca Rare Disease, and Novartis Pharmaceuticals; she is also the principal investigator at Novartis Pharmaceutical; has served as a consultant for Dianthus Therapeutics and Aurinia Pharmaceuticals and as a Principal Investigator for Apellis Pharmaceuticals; and receives royalties from UpToDate. RDS has received research funding from Veloxis and Merck, reported equity in Pfizer, and received royalties from UpToDate. DCB has received research funding from CareDx; served as a paid consultant for CareDx, Medeor Therapeutics, and Sanofi; and received royalties from UpToDate and Transplantation. JPA is part of the Scientific Advisory Board of Complement Corporation and Kypha, Inc., Scientific Advisory Board. Furthermore, he has served as a consultant for Celldex Therapeutics, formerly Avant Immunotherapeutics, Inc., Biothera, and Clinical Pharmacy Services, CDMI. All the other authors declared no conflicting interests.

## Funding

This work was supported by the Foundation of Barnes Jewish Hospital.

## Data Availability Statement

The data used in this study were from patient records and are not publicly available because of restrictions as they contain information that could compromise the privacy of the participants. Their use requires institutional review board (IRB) approval from the requestor and the Washington University IRB, in addition to a data use agreement. The authors will consider requests for the disclosure of clinical study participant-level data (beyond the details already provided in the Supplementary Tables) and supporting documents such as statistical analyses, provided that participant privacy is assured through methods such as data deidentification or anonymization (as required by applicable law). We are open to collaborating with other academic investigators.

## Supplementary Material

Supplementary File (PDF) **Table S1.** Detailed information, including age at transplant, etiology of end-stage kidney disease, genetic variants, immunosuppression regimen, and allograft function, were obtained for the 68 cases. **Table S2.** Multivariate analysis for death-censored graft failure (DCGF; group 1). **Table S3.** Multivariate analysis for death with functioning graft (DWFG; group 1). **Table S4.** Multivariate analysis for recurrent thrombotic microangiopathy (group 1).

## Footnotes

Supplementary File (PDF)
**Table S1.** Detailed information, including age at transplant, etiology of end-stage kidney disease, genetic variants, immunosuppression regimen, and allograft function, were obtained for the 68 cases. **Table S2.** Multivariate analysis for death-censored graft failure (DCGF; group 1). **Table S3.** Multivariate analysis for death with functioning graft (DWFG; group 1). **Table S4.** Multivariate analysis for recurrent thrombotic microangiopathy (group 1).
