# Supplementary material for: Thrombotic Microangiopathy After Kidney Transplantation: Insights Into Genetic Etiology and Clinical Outcomes
Source: Kidney Int Rep. 2025 Jan 30;10(4):1152–62. doi: 10.1016/j.ekir.2025.01.026 (PMC12034877; doi:10.1016/j.ekir.2025.01.026)
Supplement: Supplementary File (PDF) — Table S1. Detailed information, including age at transplant, etiology of end-stage kidney disease, genetic variants, immunosuppression regimen, and allograft function, were obtained for the 68 cases. Table S2. Multivariate analysis for death-censored graft failure (DCGF; group 1). Table S3. Multivariate analysis for death with functioning graft (DWFG; group 1). Table S4. Multivariate analysis for recurrent thrombotic microangiopathy (group 1). [file mmc1.pdf]

Supplementary Table S1

Patients	Age at transplant (yrs)	Etiology of ESKD	Genetic Testing	Kidney Transplant	Immunosuppression	Allograft function (Cr (mg/dl; UPC mg/g) as of April 2023
1	38	aHUS	Pathogenic FH (Cys611Stop)	DDKT	Myfortic, tacrolimus, prednisone and eculizumab	1.6; 400
2	47	aHUS	Pathogenic FH (c.619+1G>A)	DDKT	Myfortic, tacrolimus, prednisone and eculizumab	1.1; none
3	28	aHUS	Pathogenic FH (c.3493+ 1 G>A)	DDKT	Myfortic, tacrolimus, prednisone and eculizumab	0.7; none
4	49	aHUS	Pathogenic FH (S1191L)	DDKT	Myfortic, tacrolimus, prednisone and ravulizumab	1.3; none
5	31	aHUS	Pathogenic FI (G162D)	1st DDKT (RTD after 2 yrs, unclear etiology, severe IFTA); 2nd DDKT	Tacrolimus, prednisone and eculizumab	1.6; 350
6	35	aHUS	Pathogenic FI (G119Stop)	1st DDKT (RTD after 3 yrs, AMR); 2nd DDKT	Myfortic, tacrolimus, prednisone and ravulizumab	0.8; none
7	55	aHUS	Pathogenic C3 (R592Q)	DDKT	Tacrolimus, prednisone and ravulizumab	1.1; none
8	39	aHUS	Pathogenic FI (E554V)	DDKT	Myfortic, tacrolimus, prednisone and eculizumab	1.5; none
9	19	HUS	VUS FI (R406H) Identified after 2nd transplant. Functional studies reclassify as likely pathogenic[12].	1st DDKT (RTD after 1 yr; ACR); 2nd DDKT (RTD after 2 yrs; AMR)	None	RTD (Awaiting 3rd transplant)
10	25	HUS	VUS C3 (Glu279Lys)[13]; Identified after 1st transplant	1st DDKT (RTD after 2 yr; ACR); 2nd DDKT	Myfortic, tacrolimus and prednisone and eculizumab	1.2; none
11	30	HUS	Likely Pathogenic THBD (P495S); Identified after 1st transplant	1st DDKT (RTD after 8 years; chronic TMA); 2nd DDKT	Myfortic, tacrolimus, prednisone and ravulizumab	1; none

Supplemen[illegible] Table S1 (continued)

Patients	Age at transplant (yrs)	Etiology of ESKD	Genetic Testing	Kidney transplant	Immunosuppression	Allograft function (Cr (mg/dl; UPC mg/g)
12	39	HTN	Likely pathogenic FB (V372I).; Identified during pre-evaluation	DDKT	Myfortic, tacrolimus, prednisone and eculizumab	1.0; 300
13	26	HTN	VUS C3 (K633R)[14,15]; Identified during pre-evaluation	DDKT	Myfortic, tacrolimus, prednisone and eculizumab	0.8; none
14	39	HTN	Compound heterozygous deletion resulting in homozygous CFHR1 deletion; FH autoAb+ (1020 AU; normal range <200 AU); Identified during pre-eval	DDKT	Myfortic, tacrolimus, prednisone and Ravulizumab	1.3; none
15	36	HTN	VUS FI (V152M): Functional studies reclassify as pathogenic[16,17]; Identified after loss of 1st allograft.	1st LUKT (nephrectomy day 12 due to thrombosis); 2nd DDKT	Myfortic, tacrolimus, prednisone and ravulizumab	1; none
16	42	HTN	FH autoAbs+ (550 AU; normal range <200 AU); Identified during pre-evaluation	DDKT	Myfortic, tacrolimus, prednisone and ravulizumab	1.8; none
17	26	HTN	VUS THBD (A43T); VUS C3 (R735W)[18] Identified post-transplant	LRKT (TMA post txp day 5)	Myfortic, tacrolimus, prednisone and Ravulizumab	3; 1500
18	34	HTN	VUS FI (P64L): functional studies reclassify as pathogenic[16,17]; Identified during pre-evaluation	DDKT	Myfortic, tacrolimus, prednisone and ravulizumab	1.2; none
19	39	HTN	Homozygous CFHR3-1 deletion; FH autoAb+ (373 AU; 650 AU; normal range <200AU); Identified after 1st transplant	LUKT (RTD FSGS/TMA in allograft after 7 years);2nd LUKT	Azathioprine, tacrolimus and prednisone. Ravulizumab (stopped after 2 years once FH autoAb undetectable)	1.2; none
20	32	HTN	THBD (C224G); Variant is novel, rare and predicted to be damaging; Identified during pre-evaluation	DDKT	Myfortic, tacrolimus, prednisone and eculizumab	1.1; None
21	61	HTN	Homozygous CFHR3-1 deletion; FH autoAb+ (775 AU; normal range <200 AU); Identified after transplant	DDKT (TMA/ATN post txp day 7)	Myfortic, belatacept, prednisone and ravulizumab	1.1; 350
22	24	HTN	FH autoAbs+ (850 AU; normal range <200 AU); Identified after 1st transplant	1st LRKT (transplant nephrectomy post-op day 1 due to thrombosis); 2nd LUKT	Myfortic, tacrolimus, prednisone and ravulizumab	1.4; none
23	28	HTN	Genetic testing not conducted for unclear reasons	LRKT (thrombosis posttxp day 1, txp nephrectomy); LUKT (RTD TMA)	None	RTD
24	43	HTN	VUS MCP (P324L) – shows decreased expression[19]; Identified during pre-evaluation	DDKT	Myfortic, everolimus and prednisone eculizumab (stopped after 6 months)	1.5; 300

Supplementary Table S1 (continued)

Patients	Age at transplant (yrs)	Etiology of ESKD	Genetic Testing	Kidney transplant	Immunosuppression	Allograft function (Cr (mg/dl; UPC mg/g)
25	41	Biopsy proven TMA	VUS Factor B (Gly17Asp)	DDKT	None	Deceased
26	45	Biopsy proven TMA	Likely pathogenic C3 (S1619R); Identified during pre-evaluation	DDKT	Myfortic, tacrolimus, prednisone and ravulizumab	1; none
27	36	Biopsy proven TMA	Likely pathogenic MCP homozygous intron IVS-78 G>A; VUS PLG (A494V); Identified after transplant	DDKT (postop thrombosis, underwent nephrectomy)	None	RTD
28	49	Biopsy proven TMA	VUS C3 (F222I); Homozygous CFHR3-1 deletion; FH autoAb+ (44 units/ml; 53 units/ml; normal range <22 units/ml)	LUKT	Tacrolimus, prednisone and eculizumab	1; none
29	26	Preeclampsia	Homozygous CFHR3-1 deletion; FH autoAb+ (1050 AU; normal range <200 AU); Identified during pre-evaluation	LUKT	Azathioprine, tacrolimus, prednisone and ravulizumab (discontinued after 1 year; FH autoAb undetectable)	1.8; none
30	47	Preeclampsia/ native kidney with infarct and necrosis	Homozygous CFHR3-1 deletion; FH autoAb+ (554 AU; normal range <200 AU) ; Identified during pre-evaluation	DDKT	Myfortic, tacrolimus and prednisone. Eculizumab stopped after 3 months (FH AutoAb undetectable; being monitored with monthly labs)	1; none
31	26	Preeclampsia	Likely benign FH (D693N)[20] Identified after transplant	1st DDKT (RTD after 2 yrs; ACR)	None	RTD
32	26	Preeclampsia	Pathogenic FI (A240G); Identified during pre-evaluation	DDKT	Myfortic, tacrolimus, prednisone and ravulizumab	1; none
33	20	Preeclampsia	VUS FH (P1051L): Reclassified as likely pathogenic based on structural mapping of the variant (unpublished AJ); Identified during pre-evaluation	DDKT	Myfortic, tacrolimus, prednisone and eculizumab	0.8; none
34	45	Preeclampsia	Homozygous CFHR3-1 deletion; FH autoAb+ (80 units/ml; normal range <22 units/ml); Identified after transplant	1st DDKT (RTD after AMR)	None	RTD
35	46	Preeclampsia	VUS FH (c.3311-3_331-2delinsGT); Re-classified as pathogenic based on low FH levels (unpublished AJ); Identified after 2nd transplant.	Lost 2 allografts to TMA	None	Deceased
36	45	Preeclampsia	Genetic testing not conducted	DDKT (RTD due to TMA)	None	RTD 4/2016

Supplementary Table S1 (continued)

Patients	Age at transplant (yrs)	Etiology of ESKD	Genetic Testing	Kidney transplant	Immunosuppression	Allograft function Cr (mg/dl); UPC mg/g)
37	35	Lupus	VUS FH (I453L): functional studies reclassify as likely pathogenic[21]. Identified after 1st txp	1st DDKT (TMA/AMR within 6 months); 2nd DDKT (AMR after 2 years)	Tacrolimus, prednisone plaquenil and eculizumab	2.2; none
38	33	Lupus	VUS FH (I372V): functional studies reclassify as likely pathogenic[21]. Identified after 2nd txp	1st DDKT (TMA, nephrectomy postop day 5); 2nd DDKT (TMA/cortical necrosis after 2 years)	None	RTD
39	35	IgA + TMA; Developed preeclampsia and TMA during post partum leading to ESKD	VUS C3 (c.-2-1insAC); identified during pre-evaluation	LUKT	Myfortic, tacrolimus, prednisone and ravulizumab	1.3; none
40	26	IgA + TMA	FH autoAb+ (57 units/ml; normal range <22 units/ml); Identified during pre-evaluation	DDKT	Myfortic, tacrolimus and prednisone. Eculizumab stopped after 3 months (FH AutoAb undetectable)	1.2; none
41	28	Membranous nephropathy	VUS FI (I357M) - functional studies reclassify as likely pathogenic[22]. Identified after txp	LUKT (recurrent membranous + TMA)	None	Deceased
42	33	ADPKD	VUS FI (I416L)[14]; identified after the 1st txp	1st DDKT (Lost to thrombosis/cortical necrosis); 2nd DDKT	Myfortic, tacrolimus, prednisone and eculizumab	1.5; none

Supplementary Table S1 (continued)

Patients	Age at transplant (yrs)	Etiology of ESKD	Genetic Testing	Kidney transplant	Immunosuppression	Allograft function (Cr (mg/dl; UPC mg/g)
43	59	ADPKD	VUS DGKE (L469P)	DDKT	Tacrolimus, Prednisone Myfortic and Eculizumab (stopped).	1.23; none
44	57	Diabetes Mellitus	No variants identified	DDKT	Belatacept, Myfortic and prednisone	1.40; none
45	61	Light chain disease	Genetic testing not done	DDKT	Belatacept, Myfortic and prednisone	1.45; none
46	61	Diabetes Mellitus	Genetic testing not done	DDKT (belatacept switched back to tacrolimus after ACR/AMR)	None	RTD and Deceased
47	73	HIV Nephropathy	Benign variant CFHR2 (E199*)	DDKT (developed Kaposi's sarcoma, belatacept changed to everolimus)	Everolimus, Myfortic and prednisone	2.6; 200
48		Diabetes Mellitus	No variants identified	DDKT (belatacept switched back to tacrolimus)	Tacrolimus, Myfortic and prednisone	2.3; 800
49	44	Diabetes Mellitus	No variants identified	DDKT	Belatacept, Myfortic and prednisone	0.8; none
50	43	FSGS	No variants identified	DDKT (was switched to belatacept and was on it at the time of RTD)	None	RTD (due to septic shock)
51	69	Lymphoma	No variants identified	DDKT	Everolimus, Myfortic, prednisone and Eculizumab (stopped)	1.86; 200
52	75	Vasculitis	Genetic testing not done	DDKT (belatacept switched back to tacrolimus after MMF was stopped due to recurrent infections)	Tacrolimus and prednisone	0.95; none
53	69	Chronic Glomerulonephritis	No variants identified	DDKT	Belatacept, Myfortic and prednisone	0.7; none
54	26	SLE	No variants identified	DDKT	Belatacept, Azathioprine and prednisone	0.9; none
55	30	HTN	No variants identified	DDKT (c/b AMR)	Belatacept, Myfortic and prednisone	2.07; none
56	48	Membranous nephropathy	No variants identified	DDKT (c/b CMV viremia and recurrent membranous nephropathy)	Belatacept, Myfortic and prednisone	1; 3500

Supplementary Table S1 (continued)

Patients	Age at transplant (yrs)	Etiology of ESKD	Genetic Testing	Kidney transplant	Immunosuppression	Allograft function (Cr (mg/dl; UPC mg/g)
57	71	TMA after a raccoon bite	No variants identified	DDKT	Tacrolimus and prednisone	1.2; none
58	41	Biopsy-proven TMA	No variants identified	DDKT	Tacrolimus, Myfortic and prednisone	1.2; none
59	26	IgA nephropathy+ TMA	No variants identified	DDKT	Tacrolimus, Myfortic and prednisone	0.8; none
60	34	Renal cortical necrosis/TMA	No variants identified	DDKT	Tacrolimus and prednisone	0.9; none
61	51	HUS	No variants identified	DDKT	Tacrolimus, Myfortic and prednisone	2.4; 0.2
62	71	FSGS	No variants identified	DDKT (c/b ACR/AMR; TMA; recurrent CMV viremia)	Tacrolimus (was switched to belatacept and everolimus for varying periods, then back on tacrolimus), Myfortic and prednisone	2.3; 400
63	30	HUS	No variants identified	DDKT	Tacrolimus, Myfortic and prednisone	1.4; none
64	68	HTN	No variants identified	DDKT (c/b TMA in the allograft; switched to belatacept and treated with ravulizumab for 3 months; stopped due to no response)	None	Deceased
65	72	Diabetes Mellitus	No variants identified	DDKT (TMA in the allograft speculated to be due to CMV viremia. Responded to treatment of infection and eculizumab)	None	Deceased
66	29	SLE	No variants identified	DDKT (c/b chronic AMR/membranous lupus/TMA; treated with eculizumab in addition to other IS for 6 months with no response)	None	RTD after chronic AMR
67	60	ADPKD	No variants identified	DDKT (c/b AMR/TMA)	Tacrolimus, Myfortic and prednisone	1.7; 300
68	51	Membranous nephropathy	No variants identified	DDKT (c/b AMR/CMV viremia/TMA)	None	RTD after chronic AMR

Supplementary Table S2

Multivariate Analysis for Death Censored Graft Failure (Group 1)						
	Univariate			Multivariate		
**Variable**	**aHR**	**95% CI**	**p-value**	**aHR**	**95% CI**	**p-value**
**Recipient Characteristics**						
Age (yr)	0.91	0.84-0.98	0.02	0.88	0.79-0.96	0.01
Male sex	0.51	0.15-1.79	0.3			
Race						
• White Non-Hispanic	0.6	0.17-2.15	0.44			
• Black Non-Hispanic	0.74	0.16-3.50	0.7			
BMI (kg/m^2)	1.02	0.93-1.13	0.64			
Pretransplant Dialysis	1.44	0.18-11.16	0.75			
HLA mismatch	1.14	0.73-2.00	0.6			
• A mismatch	3.014	1.14-10.37	0.04	4.42	1.41-18.02	0.02
• B mismatch	0.99	0.42-2.89	0.99			
• DR mismatch	0.977	0.36-2.62	0.96			
Preformed DSA	0.44	0.10-2.07	0.3			
Living Donor	0.89	0.23-3.39	0.86			
• Related vs unrelated	1.31	0.16-10.90	0.8			
**Donor Characteristics**						
Age (yr)	0.987	0.94-1.04	0.61			
Male sex	1.55	0.41-5.89	0.52			
BMI	1.05	0.97-1.11	0.16			
Race						
• White Non-Hispanic	0.62	0.16	2.4			
• Black Non-Hispanic	0.62	0.08-4.90	0.65			
**Transplant Characteristics**						
Cold ischemia time	1.02	0.96-1.08	0.59			
DGF	1.35	0.29-6.33	0.7			

Supplementary Table S3

Multivariate Analysis for Death With Functioning Graft (Group 1)						
	Univariate			Multivariate		
**Variable**	**aHR**	**95% CI**	**p-value**	**aHR**	**95% CI**	**p-value**
**Recipient Characteristics**						
Age (yr)	1.02	0.94-1.09	0.6			
Male Sex	3.04	0.34-29.27	0.34			
Race<br>• White Non-Hispanic<br>• Black Non-Hispanic	 0.17<br>6.31	 0.15-1.99<br>0.56 - 70.6	 0.16<br>0.13			
BMI (Kg/m^2)	1.02	0.84-1.24	0.98			
Pretransplant Dialysis	0.77	0.06-9.38	0.84			
HLA mismatch	0.789	0.43-1.63	0.47			
• A mismatch	0.86	0.23-1.16	0.81			
• B mismatch	0.9	0.21-6.12	0.9			
• DR mismatch	0.29	0.031-2.08	0.23			
Preformed DSA	3.89	0.35-43.04	0.27			
Living Donor	6.59	0.66-65.66	0.1			
• Related vs Unrelated	2.15	0.1343-34.50	0.59			
**Donor Characteristics**						
Age (yr)	1.015	0.92-1.1	0.75			
Male sex	1.26	0.11-13.95	0.85			
BMI (Kg/m^2)						
Race						
• White Non-Hispanic	1.03	0.09-11.40	0.98			
• Black Non-Hispanic	1.2	0.10 - 14.07	0.89			
**Transplant Characteristics**						
Cold ischemia time	0.94	0.77-1.06	0.39			
DGF	1.25E-08	0	0.99			

Supplementary Table S4

Multivariate Analysis for Recurrent TMA (Group 1)

	Univariate			Multivariate		
**Variable**	**OR**	**95% CI**	**p-value**	**OR**	**95% CI**	**p-value**
**Recipient Characteristics**						
Age (yr)	0.94	0.87-1.017	0.08			
Male Sex	1.225	0.32-4.74	0.77			
Race						
• White Non-Hispanic	1.5	0.33-6.80	0.59			
• Black Non-Hispanic	0.47	0.08-2.57	0.38			
BMI (Kg/m^2)	0.98	0.89-1.09	0.7			
Pretransplant Dialysis	1.69	0.1693-16.91	0.64			
**HLA mismatch**	1.38	0.77-2.46	0.24			
• A mismatch	2.14	0.73-6.27	0.17			
• B mismatch	2.13	0.58-7.78	0.22			
• DR mismatch	1.4	0.48-4.10	0.54			
Preformed DSA	0.35	0.0637-1.872	0.22			
Living Donor	6.99	1.57-31.25	0.01			
Donor Related	5.8	0.47-71.06	0.17			
**Donor Characteristics**						
Age (yr)	1.03	0.98-1.08	0.27			
Male Sex	1	0.24-4.14	1			
BMI	2.83	0.06-1.09	0.98			
Race						
• White Non-Hispanic	1	0.17-6.03	1			
• Black Non-Hispanic	1.8	0.26-12.41	0.55			
**Transplant Characteristics**						
Cold ischemia time	0.94	0.86-1.02	0.11			
DGF	2.16	0.40-11.60	0.37			

ESKD, End stage kidney disease; aHUS, atypical hemolytic uremic syndrome; FH, factor H; FI, factor I;VUS, variant of uncertain significance; THBD, thrombomodulin; DDKT, deceased donor kidney transplant; RTD, return to dialysis; IFTA interstitial fibrosis tubular atrophy; ACR, acute cellular rejection; AMR, antibody-mediated rejection; TMA, thrombotic microangiopathy; UPC, urine protein creatinine ratio; Cr, creatinine; HTN, hypertension; LUKT, living unrelated kidney transplant; FSGS, focal segmental glomerulosclerosis; LRKT, living related kidney transplant; ATN, acute tubular necrosis; MCP, membrane cofactor protein; ADPKD, autosomal dominant polycystic kidney disease; DGKE, diacylglycerol kinase epsilon; c/b, complicated by; SLE, systemic lupus erythematosus; CMV, cytomegalovirus; BMI, body mass index; DGF, delayed graft function; CI, confidence interval; DSA, donor specific antibody;
